
JOURNAL INFORMATION
==============================
NLM Title Abbreviation: ScientificWorldJournal
Journal ID: ScientificWorldJournal
NLM Journal ID: 101131163
Journal ID: TSWJ

The Scientific World Journal

ISSN: 2356-6140
EISSN: 1537-744X
Publisher: Wiley

ARTICLE INFORMATION
==============================
PMCID: PMC6083914
PMID: 30147496
DOI: 10.1100/tsw.2001.143
Article ID: 123719
Article version: 1
Subjects: Short Report

Regulation of Ciap1 and Xiap Expression by Nitric Oxide, Cytokines, and Its Relation to Apotosis Induction in Rat Mesangial Cells and Raw 264.7 Macrophages

Manderscheid Markus *
Meszligmer Udo K.
Pfeilschifter Josef
pharmazentrum, Klinikum der Johann Wolfgang Goethe-Universität Frankfurt am Main, Theodor-Stern-Kai 7, D-60590 Frankfurt am Main, Germany
*Markus Manderscheid: manderscheid@em.uni-frankfurt.de
Publication date: 2001
Electronic publication date: 2001 Sep 24
Volume: 1
First page: 43
Last page: 43
Copyright: Copyright © 2001 Markus Manderscheid et al.
Copyright year: 2001
License: This is an open access article distributed under the Creative Commons Attribution License, which permits unrestricted use, distribution, and reproduction in any medium, provided the original work is properly cited.
License URL: https://creativecommons.org/licenses/by/3.0/

Funding: Deutsche Forschungsgemeinschaft SFB553

==============================
Miami *Nature Biotechnology* Short Reports
*TheScientificWorld* (2001) 1(S3), 43SR
ISSN 1532-2246; DOI 10.1100/tsw.2001.143

### REGULATION OF CIAP1 AND XIAP EXPRESSION BY NITRIC OXIDE, CYTOKINES, AND ITS RELATION TO APOPTOSIS INDUCTION IN RAT MESANGIAL CELLS AND RAW 264.7 MACROPHAGES

Markus Manderscheid*, Udo K. Meßmer, and Josef Pfeilschifter

*pharmazentrum*, Klinikum der Johann Wolfgang Goethe-Universität Frankfurt am Main, Theodor-Stern-Kai 7, D-60590 Frankfurt am Main, Germany
* Manderscheid@em.uni-frankfurt.de

**INTRODUCTION.** Mesangial cells and RAW 264.7 macrophages respond to different NO donors within 16-24h or 6-8h, respectively, with apoptotic cell death (1). RAW 264.7 macrophages also die in response to endogenous NO production. In contrast, endogenous NO production failed to significantly induce cell death in mesangial cells (2,3). We hypothesized that differences in the expression of antiapoptotic proteins in particular the inhibitor of apoptosis (IAP) protein family might be responsible for this cell type-specific behaviour.

**METHOD.** To this end, by performing RNase Protection Assay and Western Blot Analysis, we compared IAP expression in relation to apoptosis induction in response to NO and cytokines in both cell types.

**RESULTS.** In mesangial cells IL-1$\beta$ and TNF-$\alpha$ induced cIAP1 mRNA expression within 3 h. In contrast, XIAP mRNA levels remained unaffected by cytokines. Although coincubation of cells with IL-1$\beta$ and TNF-$\alpha$ or IL-1$\beta$ and bFGF resulted in a synergistic induction of iNOS, comparable potentiating effects were absent regarding cIAP1 induction. Also exogenously released NO from NO donors promoted cIAP1 mRNA upregulation in mesangial cells whereas XIAP mRNA was downregulated. However, these changes seen on the mRNA level were not adequately translated onto the protein level and corresponding values for cIAP1 and XIAP were only slightly affected. In contrast, in LPS/interferon-$\gamma$-stimulated RAW 264.7 macrophages, we found a massive NO-dependent downregulation of cIAP1 and XIAP protein level that correlated temporally with induction of apoptosis. This effect was at least partially reversed by L-NMMA, an inhibitor of NOS activity.

In summary, in macrophages we found a direct correlation between the downregulation of IAP protein levels and the induction of apoptosis by endogenous NO. By contrast, a stable level of IAP protein in mesangial cells might represent a mechanism constituting resistance of the cells against endogenously produced NO.

**ACKNOWLEDGEMENT.** This work was supported by a grant of the Deutsche Forschungsgemeinschaft (SFB553).

**REFERENCES.**

1. Meßmer, U.K., Lapetina, E.G., and Brüne, B. (1995) Mol. Pharmacol. 47, 757-765
2. Sandau, K., Pfeilschifter, J., and Brüne, B. (1997) J. Immunol. 158, 4938-4946
3. Nitsch, D.D.,Ghilardi, N., Muhl, H., Nitsch, C., Brune, B., Pfeilschifter, J., et al. (1997) 4. Am. J. Pathol. 150 (3), 889-900
